# Evaluation of the Spies™ modalities image quality

**DOI:** 10.1590/S1677-5538.IBJU.2016.0324

**Published:** 2017

**Authors:** Esteban Emiliani, Michele Talso, Mohammed Baghdadi, Aarón Barreiro, Andrea Orosa, Pol Serviàn, Pavel Gavrilov, Silvia Proietti, Olivier Traxer

**Affiliations:** 1 Department of Urology, Hôpital Tenon, Université Pierre et Marie Curie - Paris VI, Paris, France;; 2 GRC lithiase (Grouped Recherche Clinique) Université Paris VI, Pierre et Marie Curie, Paris, France

**Keywords:** Ureteroscopy, Diagnosis, Lithotripsy, Technology

## Abstract

**Introduction:**

The Spies™ system (Karl-Storz®) was introduced into digital ureteroscopy to improve endoscopic vision. To date, there is no data to either indicate which of the Spies modalities is better for improving diagnosis and treatment procedures, nor to compare the modalities in terms of image quality. The aim of this study was to evaluate and compare the image quality of five Spies™ modalities (SM) to the standard white light in an in-vitro model.

**Materials and Methods:**

Two standardized grids and 3 stones of different composition were recorded in white light and the 5SM (Clara, Chroma, Clara+Chroma), Spectra A and B) using 4 standardized aqueous scenarios. Twelve templates were done in order to simultaneously compare the same objective in the different modalities. Six urologists, five medical students, five urology residents, and five persons not involved with urology evaluated each video on a scale of 1 (very bad) to 5 (very good).

**Results:**

Comparing white light to SM, subjects scored better the quality of Clara and Clara+Chroma than white light (p=0.0139 and p<0.05) and scored worse Spectra A and B (p=0.0005 and p=0.0023)). When comparing Clara to the other SM, it was ranked equivalent to Clara+Chroma (p=0.67) and obtained a higher rank than Chroma, Spectra A and B (p<0.05, p=0.0001 and p=0.0001). In the multivariate analysis mean scores were higher among urologists.

**Conclusion:**

In all analyzed scenarios, the subjects ranked Clara and Clara+Chroma as the modalities with better image quality compared to white light.

## INTRODUCTION

Since the arrival of digital ureteroscopy, several new technologies have been used to improve endoscopic vision. Examples of such technologies include the NBI™ system (Olympus®) (1); the photodynamic diagnosis (2) or the Storz Professional Image Enhancement System: Spies™ system (Karl-Storz®, Tuttlingen, Germany) integrated in the Karl-Storz® FlexXC™ ureteroscope that uses five different modalities of visual enhancement besides the standard white light. This system captures an image in white light through a red, green and blue (RGB) camera and performs a digital reprocessing to modify and generate the new image modality desired (3).

To date there is no evidence regarding which of the five modalities is better to improve diagnosis or treatment procedures, nor data comparing the modalities in terms of image quality; also, the company does not recommend its use for any specific situation (4).

The aim of this study was to evaluate and compare the image quality of the five Spies™ modalities (SM) to the standard white light in an in-vitro model.

## MATERIALS AND METHODS

The Spies™ system, integrated in the FlexXC™ ureteroscope, uses five different modalities of visual enhancement to improve tumor diagnosis. Aside from the standard white light it uses the following modalities: Spectra A and B by color spectral separation using different color filter settings that allow better contrast between tissues, Clara: by manipulating the image brightness to achieve better views of dark spots, Chroma by increasing color contrast and Clara+Chroma by combining both (3).

To evaluate the image quality, two standardized grids or test patterns of colors and resolution specifically designed to test image quality (Edmund Optics, Barrington, NJ®) (5, 6) and 3 stones of different composition (monohydrate calcium oxalate, dehydrate calcium oxalate and uric acid) were used in 4 different standardized scenarios using the K-box™ simulator (Coloplast®): 110cc of saline solution, 110cc of sterile water, 110cc of saline solution mixed with 20cc of pure contrast and 110cc of saline solution mixed with 3cc of iodine solution 0.3%. A total of 72 videos were made after recording the three objects in all of the six modalities in the four scenarios. To conserve the image quality, the videos were made in high definition with a calibrated Karl-Storz® recording device.

**Figure 1:**
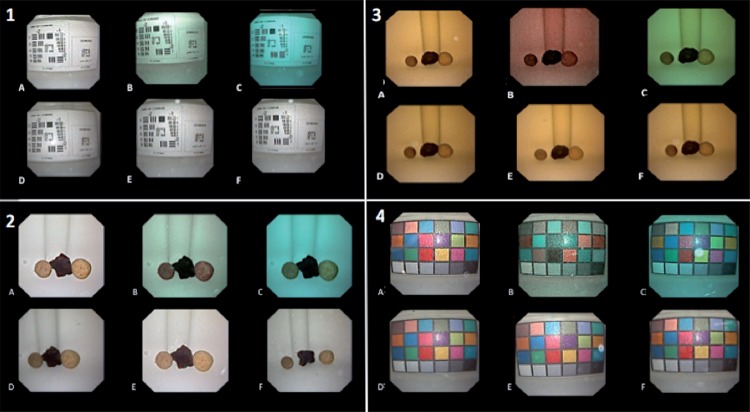
Example of the templates used to evaluate the same object in the different scenarios.

Twelve templates were done randomizing the position of the videos to simultaneously compare the same objectives recorded in the different modalities (Figure-1) in order to perform an absolute scale of merit from 1 (very bad) to 5 (very good).

1) Sharpness grid template in the different Spies modes in saline solution (a: White light, b: Clara, c: Chroma, d: Clara+Chroma, e: Spectra A, f: Spectra B).

2) Stone view template in the different Spies modes in Sterile Water (a: White light, b: Clara, c: Chroma, d: Clara+Chroma, e: Spectra A, f: Spectra B).

3) Stone view template in the different Spies modes in saline solution mixed with 3 cc of iodine solution. (a: White light, b: Clara, c: Chroma, d:Clara+Chroma, e: Spectra A, f: Spectra B).

4) Color grid template in the different Spies modes in saline solution with contrast. (a: White light, b: Clara, c: Chroma, d: Clara+Chroma, e: Spectra A, f: Spectra B)

Image quality was measured subjectively as in real endoscopy. A random group of six urologists, five medical students, five urology residents, and five persons not involved with the ureteroscopic procedure evaluated each video and ranked the image quality. Subjects were asked to rate perceived image degradation, sharpness of the objects, and presence of artifacts that could distort the image. Scores were tabulated by the sum of the 1-5 score for each image with each solution.

Statistical analysis was performed with the STATA 13.0 software. A T Student’s test and multivariate logistic regression analysis was employed to compare Spies™ versus standard white light as control. P<0.05 was considered statistically significant.

## RESULTS

Eight females and 13 males evaluated the videos; the mean age was 37 years (22-56). Eleven subjects had a refractive error corrected with either glasses or contact lenses. The groups were homogeneous in terms of gender, age, profession and refraction errors. The mean score in terms of image quality for each modality was: White light: 40, Clara: 46, Chroma: 41, Clara+Chroma: 45, Spectra A: 29 and Spectra B: 31. When comparing white light with the SM the subjects scored better the quality of Clara and Clara+Chroma (p=0.0139 and p<0.05 respectively) and worse Spectra A and B (p=0.0005 and p=0.0023 respectively). When comparing Clara (the modality best ranked) and the other SM, the former was equivalent to Clara+Chroma (p=0.67) and obtained a higher rank than Chroma, Spectra A and B (p<0.05, p=0.0001 and p=0.0001 respectively). Results are summarized in Table-1.

**Table 1:** Scores of all modalities.

Modality	Mean score	Score range	*White light vs. Spies Modalities	**Clara vs. Spies Modalities
White light	40	(19-60)		
Clara	46	(24-60)	p=0.0139	
Chroma	41	(19-60)	p=0.94	p < 0.05
Clara+Chroma	45	(29-60)	p< 0.05	p=0.6767
Spectra A	29	(12-58)	p=0.0005	p=0.0001
Spectra B	31	(12-58)	p=0.0023	p=0.0001

* Comparison between the Spies modes and white light

** Comparison between the Spies modes and Clara (The Spies mod best ranked)

In the subgroup where the quality of the view of the stones was ranked, Clara and Clara+Chroma modalities were ranked as the best, being better rated than white light (p=0.0001 and p=0.0001 respectively). The image quality of the stone video in Spectra A and B had a worse scoring than white light (p=0.0055 and p=0.0052 respectively). There were no statistical differences between the aqueous scenarios using the same SM (p>0.05). Results are summarized in Table-2.

**Table 2:** Scores of all modalities when qualifying the stone images. Comparison between the Spies modes and white light.

Modality	Mean score	Score range	White light vs. Spies Modalities
White light	13	(4-20)	
Clara	17	(10-20)	p=0.0001
Chroma	13	(4-20)	p=1
Clara+Chroma	16	(10-20)	p=0.0001
Spectra A	10	(4-19)	p=0.0055
Spectra B	19	(4-20)	p=0.0052

In the multivariate analysis stratified by profession into urologist/residents and non-urologists (students, other), the mean scores were higher among the urologists (45 vs. 31, respectively). There were no differences between groups in terms of gender, corrected view or age.

## DISCUSSION

Digital ureteroscopy has brought diverse advantages for diagnostic and treatment procedures. Aside from a clear improvement in the image quality when compared to fiber optics, it has shown a significant reduction of operative times when treating stones (7).

Another benefit of digital ureteroscopy is a set of the novel integrated tools that have been developed to enhance ureteroscopic visualization through light absorption by increasing brightness and contrast or color spectral separation.

As conservative treatment can be offered to patients with upper urinary tract carcinoma with low grade, non-invasive and small tumors (8), there is an increasing interest in developing image enhancement machinery integrated into flexible ureteroscopes. The challenge of adequate diagnosis arises in cases of doubtful small and flat lesions where radiological and cytological evaluations may have low accuracy (3, 9, 10). For these situations, aside from Spies™, the NBI™ system (Olympus®) was developed specifically to increase tumor diagnosis accuracy, contrary to Spies™ in which the company does not recommend its use for any specific situation (4). In the upper urinary tract NBI™ system has initially demonstrated improved tumor detection rates by 22.7% compared to white light, however further evaluation is needed in order to recommend its daily clinical use (1, 11, 12). Likewise, to our knowledge there are no studies regarding the upper urinary tract tumor diagnosis with Spies™.

The SM best ranked overall was Clara and Clara+Chroma compared to white light and the other SM. Clara manipulates the image brightness, Chroma intensifies color contrast and Clara+Chroma combines both. As Chroma and Clara+Chroma theoretically increase sharpness (which means a more detailed image boundary, sharp and not blurred) an image with better quality may be perceived and could explain why it was considered better than the other modalities.

Spectra A and B looks for tissue differentiation by filtering color spectra. Spectra A filters red to remove the base redness of urothelium while gains contrast in the remaining colors. Spectra B decreases red spectrum while increasing the green and blue for the same purpose. Although a color grid was used to record the videos, the color accuracy was not evaluated as the two SMs are intended to modify it. In this study the image quality of Spectra A and B had the worse score compared to white light and the other SM (In both grid and stones evaluation). Although the system takes high definition images the digital manipulation of an image may decrease the image quality. This could explain the low image quality assessment, as in this process some distortion or artifacts may be seen in the image. As the Spectra modes are commonly used to compete with other technologies for tumor diagnosis, according to these results, further in vivo studies are needed to assess whether this image quality deterioration may decrease the probability of tumor diagnosis and/or stone treatment.

In the multivariate analysis stratified by profession into urologist/residents and non-urologists (students, other), the mean scores were higher among the urologists (45 vs. 31 respectively). The subjective perception of urologists based on personal experiences and the knowledge of fiber optic and digital scopes may influence this decision. Knowing the surgical intention of the image and what surgical skills could be achieved with it, even if the image is not impeccable could increase the evaluation points.

Further, presently there is an increasing amount of urologists that use the SM as a working device for tumor ablation and stone laser treatment as surgeons may feel more confortable with the new endoscopic vision of the object to treat. This is the reason why stones of different components were evaluated. Our findings in the subgroup where the quality of the view of the stones was ranked may suggest that Clara and Clara+Chroma may be the best option for this purpose. This follows the company’s concept that this tool can also be used to achieve a better image for treatment purposes.

A limitation of this study is that it was not initially intended to describe image quality in tumors specifically, but to give an overall evaluation of the quality of the system and to explore other possible uses. This preliminary study provides information for further in vivo assessments to evaluate whether the use of Spies™ may increase the effectiveness of endoscopic procedures including ureteroscopy, cystoscopy and percutaneous nephroscopy; either for stones or tumor treatments by increasing image quality.

## CONCLUSIONS

In this in vitro study Clara and Clara+Chroma were ranked as the best Spies™ modalities with better image quality compared to white light or other Spies™ modalities. Spectra A and B had the lowest rates in all scenarios analyzed.
